# Triple-Regimen of Vemurafenib, Irinotecan, and Cetuximab for the Treatment of BRAF^V600E^-Mutant CRC: A Case Report and Review

**DOI:** 10.3389/fphar.2021.795381

**Published:** 2021-12-16

**Authors:** Su Min Cho, Abdullah Esmail, Maen Abdelrahim

**Affiliations:** ^1^ Department of Medicine, Houston Methodist Hospital, Houston, TX, United States; ^2^ Section of GI Oncology, Department of Medical Oncology, Houston Methodist Cancer Center, Houston, TX, United States; ^3^ Cockrell Center of Advanced Therapeutics Phase I Program, Houston Methodist Research Institute, Houston, TX, United States; ^4^ Weill Cornell Medical College, New York, NY, United States

**Keywords:** BRAF, CRC, Vemurafenib, Irinotecan, Cetuximab, Metastasis, SWOG, Immunotherapy, Chemotherapy

## Abstract

Mutation of the BRAF proto-oncogene is found in approximately 10% of colorectal cancers (CRC), with much of the mutation conferred by a V600E mutation. Unlike other CRC subtypes, BRAF-mutant CRC have had relatively limited response to conventional therapies and overall poor survival. We present the case of a 75-year-old man with severe nonischemic cardiomyopathy on a LifeVest who was found to have a transverse colonic mass with widespread hepatic metastatic disease and was subsequently found to have BRAF^V600E^-mutant CRC (MSI High/dMMR). After a failed therapy with FOLFOX and pembrolizumab, the patient was started on a regimen of vemurafenib, irinotecan, and cetuximab (VIC) based on the SWOG 1406 trial which had shown improved progression-free survival and response rate for the treatment of BRAF^V600E^-mutant metastatic CRC. After 40 cycles of VIC, the patient attained complete response and is in remission off chemotherapy with significant improvement. This case highlights the effectiveness of the triple-regimen of vemurafenib, irinotecan, and cetuximab as a treatment option for BRAF^V600E^-mutant CRC, which is a treatment regimen based on the SWOG 1406 trial, and also demonstrates the synergistic role of BRAF^V600E^ inhibitors and EGFR inhibitors in the treatment of BRAF^V600E^-mutant CRC.

## Introduction

Colorectal cancer (CRC) is one of the leading causes of death in the world. In the United States alone, it represents the third most common cancer mortality with up to 53,200 deaths in 2020 (Biller and Schrag, 2021).

Up to a third of patients initially present with stage IV metastatic disease at the time of diagnosis, requiring systemic therapy for chance of cure (Zacharakis et al., 2010). 5-Fluorouracil was the first chemotherapy which proved effective against CRC, and new therapeutic agents and regimens have since been adopted (Duschinsky et al., 1957; Heidelberger et al., 1957; Biller and Schrag, 2021).

The mitogen-activated protein kinases (MAPK) is a chain of kinases and proteins that relay extracellular signals via a biochemical cascade to influence essential intracellular processes such as growth, differentiation, migration, and apoptosis (Dhillon et al., 2007). A dysregulation of this signal cascade can therefore lead to uncontrolled growth and proliferation, leading to tumorigenesis (Ascierto et al., 2012). BRAF is one of the protein kinases that is frequently mutated, perhaps due to fewer mutations necessary for its constitutive activation (Michaloglou et al., 2008). Mutation of the BRAF proto-oncogene is found in approximately 10% of colorectal cancers (CRC), with much of the mutation conferred by a V600E mutation (Kopetz et al., 2019). BRAF-mutant CRCs pose a special challenge for oncologists due to their relatively limited response to conventional therapies and overall poor survival (Kopetz et al., 2019).

Greater understanding of the pathogenesis of CRC has paved the way to numerous therapies, ranging from chemotherapy to targeted therapies. Currently, irinotecan combined with cetuximab is one of the regimens approved for treatment of metastatic CRC (Kopetz et al., 2017; Kopetz et al., 2021). On April 8, 2020, the Food and Drug Administration (FDA) approved encorafenib in combination with cetuximab for the treatment of patients with metastatic CRC with BRAF^V600E^ mutation after a positive Phase III trial (Kopetz et al., 2019). In addition, an older randomized clinical trial called SWOG 1406 showed that the addition of vemurafenib to the irinotecan/cetuximab regimen showed improved progression-free survival and response rate for the treatment of BRAF^V600E^-mutant metastatic CRC (Kopetz et al., 2017; Kopetz et al., 2021). Here we present a case highlighting the effectiveness of the triple-combination therapy of vemurafenib, irinotecan, and cetuximab in the treatment of metastatic BRAF^V600E^-mutant CRC. This regimen, based on the SWOG 1406 trial, demonstrates a potential new option for treating metastatic BRAF^V600E^-mutant CRC. We also explore other potential treatment options currently under study, which reveal the synergistic role of an EGFR inhibitor and a BRAF^V600E^ inhibitor.

### Case Presentation

We present the case of a 75-year-old man with severe nonischemic cardiomyopathy on LifeVest who initially presented on December 2017 with progressive abdominal pain for 2 months. The patient was initially suspected as having a gastric ulcer by his primary care physician and was therefore referred to a gastroenterologist for endoscopic evaluation. Upper endoscopy however was only positive for a hiatal hernia. The patient’s abdominal pain continued to worsen, and he developed additional symptoms nausea, decreased appetite, fatigue, constipation. The patient visited the emergency room given the progress nature of his symptoms. CT imaging showed a circumferential narrowing of the mid-transverse colon with widespread hepatic metastatic disease. Subsequent colonoscopy showed a fungating, infiltrating, and ulcerated partially obstructing mass in the transverse colon with subsequent biopsy revealing poorly differentiated invasive adenocarcinoma on January 2018. Molecular testing was positive for BRAF-V600E mutation. Mismatch repair protein analysis was notable for loss of expression of MLH1 and PMS2. Microsatellite instability analysis was MSI-High, with instability of microsatellite markers NR-21, BAT-26, BAT-25, NR-24, and MONO-27.

The patient was started on palliative FOLFOX and was then started on bevacizumab during the third cycle of FOLFOX. This regimen was chosen due to initial concerns for GI perforation and overall poor performance status. CT chest/abdomen/pelvis was obtained every 3 months to assess therapy response. However, subsequent imaging showed progression of disease despite four cycles of FOLFOX and two cycles of bevacizumab. The treatment regimen was switched to pembrolizumab, but disease progression was still seen despite three cycles of pembrolizumab.

**FIGURE 1:**
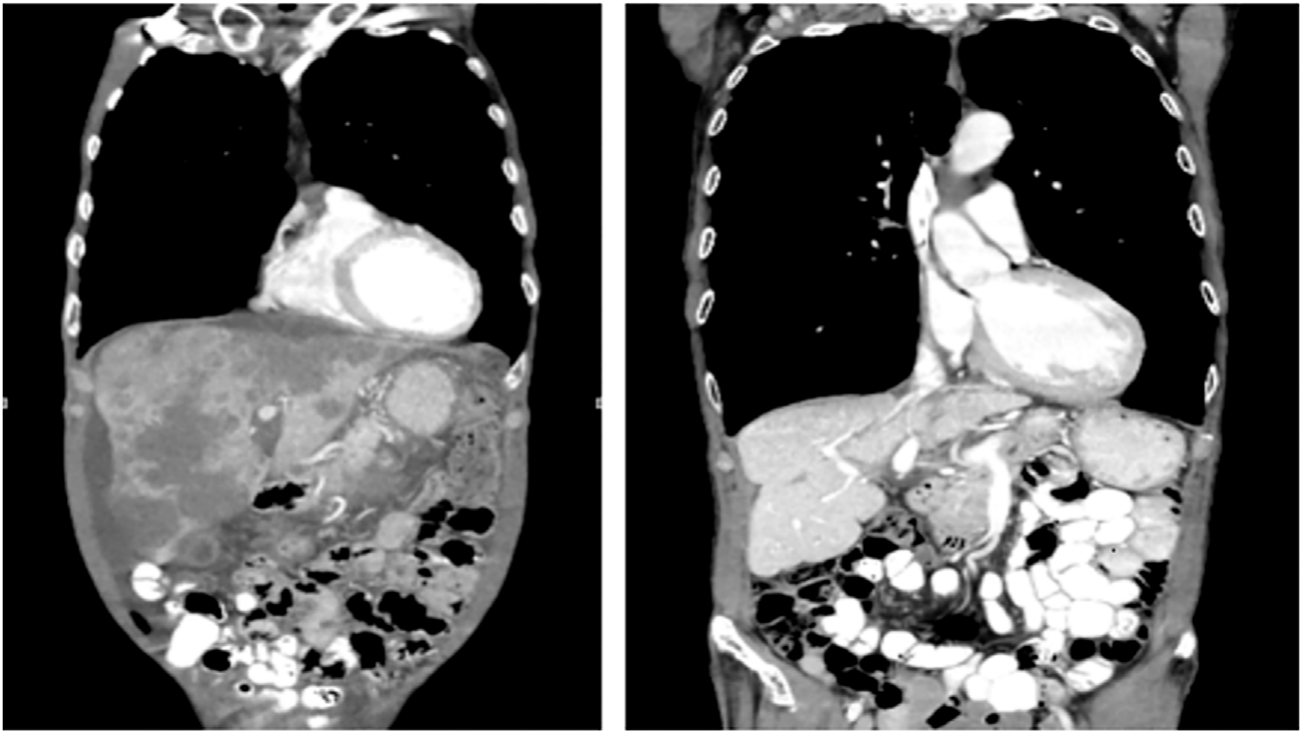
Extensive hepatic metastasis present before initiation of VIC **(left)**. In remission after 40 cycles of VIC **(right)**.

**FIGURE 2:**
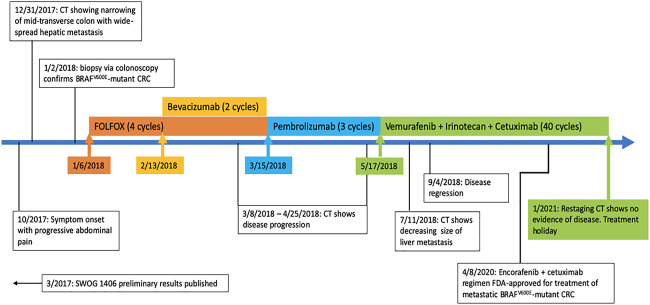
Timeline of management of metastatic BRAF^V600E^-mutant CRC (colorectal cancer).

In May 2018 the patient was started on a triple-combination therapy consisting of vemurafenib, irinotecan, and cetuximab (VIC), which was based on the SWOG 1406 clinical trial (Kopetz et al., 2017). This decision was made based on his prior therapy failures as well as the promising results of the SWOG 1406 clinical trial (Kopetz et al., 2017). At this point in time, the regimen of encorafenib and cetuximab had not yet been FDA approved (Kopetz et al., 2019). Extensive discussion was held with the patient and family regarding therapy strategy and side-effect profile, and ultimately they agreed to the experimental therapy with continued goal of palliative therapy. The dosing and frequency was modeled after that of the SWOG 1406 clinical trial (Kopetz et al., 2017; Kopetz et al., 2021). The patient received IV cetuximab at a dose of 500 mg/m^2^ (800 mg for our patient) followed by IV irinotecan at 180 mg/m2 (288 mg for our patient) on days 1 and 15, with courses repeated every 28 days (Kopetz et al., 2017; Kopetz et al., 2021). The patient also received PO vemurafenib 960 mg twice daily on days 1 through 28, with a single cycle consisting of 28 days. After five cycles of VIC therapy, the patient’s metastatic lesions of the liver had significantly decreased in size. CT imaging was continually obtained every 3 months, and each additional imaging showed either stable disease or disease regression. After 40 cycles of VIC, the patient attained complete response and is in remission off chemotherapy with significant improvement in his weight, performance status, and quality of life (Figure 1, Figure 2 **).** As of today October 2021 the patient has remained disease-free off of chemotherapy for 9 months without obvious disease recurrence, but continued surveillance CT imaging will still be performed to monitor for recurrence.

Of note, before the patient was started on VIC, the patient had been followed by a cardiologist for his nonischemic cardiomyopathy with a severely depressed LVEF 20–24%. The patient was not a candidate for an automatic implantable cardioverter defibrillator (AICD) and therefore had been on a LifeVest. Given the overall poor outlook from his metastatic disease, the patient had decided to discontinue his LifeVest for improved quality of life at the risk of a sudden cardiac death. However, after attaining complete response to VIC, the patient was able to undergo implantation of a cardiac resynchronization therapy with defibrillation (CRT-D) device with significant improvement to his functional status. The patient and his family have been very happy with the care they received in the hospital and the clinic.

## Discussion

Vemurafenib is a selective BRAF^V600E^inhibitor that had been approved by the FDA in 2011 for treatment of BRAF^V600E^-mutant metastatic melanoma, though it had limited efficacy when used as monotherapy against CRC (Korphaisarn and Kopetz, 2016). Irinotecan, a topoisomerase I inhibitor, was approved for treatment of metastatic CRC refractory to 5-FU in 1996 and is now a key drug for treatment of metastatic CRC given the survival advantage of irinotecan-based regimen (Fujita et al., 2015). Cetuximab is a monoclonal antibody against the epidermal growth factor receptor (EGFR) inhibitor. A 2004 clinical trial showed clinically-significant activity of cetuximab when given alone or in combination with irinotecan for irinotecan-refractory metastatic CRC, with greater ORR and PFS for the combination therapy as opposed to the monotherapy (Cunningham et al., 2004).

For our patient, we have chosen the triple therapy of VIC (vemurafenib + irinotecan + cetuximab) because at the time of therapy initiation (2018) the doublet regimen of encorafenib and cetuximab, based on the positive phase III trial in 2019 (8), had not yet been FDA approved. Our patient was started on VIC in 2018, and the preliminary results of the randomized phase II SWOG 1406 clinical trial published in 2017 had shown clinically-significant benefit of adding vemurafenib to the combined irinotecan/cetuximab therapy (Kopetz et al., 2017). The SWOG 1406 trial was comprised of 106 participants who had previously received 1 or 2 prior regimens except for anti-EGFR agents (Kopetz et al., 2017; Kopetz et al., 2021). Compared to the doublet irinotecan/cetuximab therapy, the triple therapy showed improved median PFS (4.4 vs 2 months) and improved response rate (17 vs 4%) (Kopetz et al., 2017; Kopetz et al., 2021). The primary conclusion of the study was that inhibition of both EGFR and BRAF combined with irinotecan was effective for the treatment of BRAF^V600E^-mutant CRC (Kopetz et al., 2017; Kopetz et al., 2021). Our patient’s PFS (36 months) has far exceeded what was shown in the study. The reason for our patient’s superior clinical response is unclear, but we suspect this was due to a distinct molecular and immunohistochemical characteristic of our patient’s cancer that was more responsive to the VIC regimen. A significant challenge was the patient’s cardiac comorbidity, namely his cardiomyopathy with an LVEF 20–24%. In view of this condition, the patient required close monitoring of his QTc (by EKG before each cycle) and volume status while receiving his chemotherapy. The overall poor prognosis had made him ineligible for an ICD, and in fact the patient’s LifeVest was discontinued during the initial stages of chemotherapy to improve his quality of life. This is no longer the case after having achieved remission, and now the patient was able to have an CRT-D device implanted.

A major strength to this case is that this case was not directly affiliated with the SWOG clinical study. We believe this allowed for a non-biased approach to our treatment regimen and assessment of the treatment response. The limitation of this case report, on the other hand, is that this is a report regarding a single patient. However, not many publications experimenting with the VIC regimen are found in the literature aside from the primary SWOG trial. One similar finding is a 2020 case report demonstrating complete response of metastatic BRAF^V600E^-mutant CRC to a regimen of fluorouracil + VIC (Wang et al., 2019). The 44 year-old patient was refractory to a regimen of capecitabine + oxaliplatin and had developed metastatic disease (Wang et al., 2019). The patient’s first cycle was irinotecan + 5-FU with subsequent cycles composed of a quad-therapy of 5-FU + VIC, and after 10 total cycles the patient had attained complete recession of metastases (Wang et al., 2019). The general conclusion of this case was similar to that of our case and the SWOG trial, aside from the concomitant usage of 5-FU along with the VIC.

The BEACON CRC phase III trial showed effectiveness of the triple regimen of encorafenib + cetuximab + binimetinib compared to the control (cetuximab + irinotecan or cetuximab + FOLFIRI) for treatment-refractory metastatic CRC (Kopetz et al., 2019). There was a clinically significant increase in median overall survival of patients who had received the triple therapy (9.0 months) compared to the control group (5.4 months) (Kopetz et al., 2019). It is worth noting that the trial had also explored the efficacy of a doublet therapy (encorafenib + cetuximab) which had a median overall survival of 8.4 months (Kopetz et al., 2019). This doublet therapy of encorafenib + cetuximab is now FDA approved for treatment of metastatic BRAF^V600E^-mutant CRC. Of note, encorafenib is a BRAF^V600E^ inhibitor just like vemurafenib which gives further gives credence to the effectiveness of utilizing both EGFR and BRAF inhibition as in like the SWOG trial.

Currently, there is research analyzing the effectiveness of the triple regimen (encorafenib + cetuximab + binimetinib) for metastatic BRAF^V600E^-mutant CRC (Grothey et al., 2020). The ANCHOR CRC trial is currently in phase II, and so far it has shown effectiveness of the afore-mentioned triple therapy as a first-line metastatic treatment, with an overall response rate of 50 and 85% of patients with decreased tumor size so far (Grothey et al., 2020). This is in contrast to the BEACON CRC trial which had enrolled patients with treatment-refractory metastatic CRC (Kopetz et al., 2019).

A recent 2018 study of 142 patients analyzed the treatment efficacy of dabrafenib + panitumumab + trametinib (D + P + T) (Corcoran et al., 2018). Dabrafenib is a BRAF^V600E^ inhibitor and panitumumab is an inhibitor of EGFR, just like vemurafenib and cetuximab are, respectively (Addeo et al., 2010; Abraham and Stenger, 2014). This study also added trametinib, a MEK inhibitor which had been FDA approved for use in BRAF^V600E/V600K^ mutated melanoma (Hoffner and Benchich, 2018). The median PFS for the D + P + T, D + P, and T + P arms were 4.2, 2.6, and 3.5 months respectively (Corcoran et al., 2018). The overall response rate was 21, 10, and 0% respectively (Corcoran et al., 2018). The median overall survival was 9.1 months, 13.2, and 8.2 respectively (Corcoran et al., 2018). In conclusion, the usage of the triple regimen of MEK + BRAF^V600E^ + EGFR inhibitors had greater overall response rate and PFS, while the dual-regimen of BRAF^V600E^ + EGFR inhibitors had the greater median overall survival.

Overall, our case highlights the effectiveness of the triple-regimen of vemurafenib, irinotecan, and cetuximab as a treatment option for BRAF^V600E^-mutant CRC, a treatment regimen based on the SWOG 1406 trial. Our case demonstrates a patient with serious cardiac comorbidity who was able to tolerate the cancer regimen and attain complete response. The current treatment trend for this metastatic BRAF^V600E^-mutant CRC at this time appears to revolve around the usage of both a BRAF inhibitor as well as an EGFR mutation.

## Data Availability

The original contributions presented in the study are included in the article/Supplementary Material, further inquiries can be directed to the corresponding author.
